# Muscle force interacts with stature to influence functionally related polar second moments of area in the lower limb among adult women

**DOI:** 10.1002/ajpa.24097

**Published:** 2020-07-31

**Authors:** Alison A. Murray, Jay T. Stock

**Affiliations:** ^1^ Department of Anthropology University of Victoria Victoria British Columbia Canada; ^2^ Department of Archaeology University of Cambridge Cambridge UK; ^3^ Department of Anthropology Western University London Ontario Canada; ^4^ Department of Archaeology Max Planck Institute for the Science of Human History Jena Germany

**Keywords:** body size, cross‐sectional geometry, muscle force, skeletal muscle

## Abstract

**Objectives:**

We sought to determine the relationships between muscle size, function, and polar second moments of area (J) at the midshaft femur, proximal tibia, and midshaft tibia.

**Materials and Methods:**

We used peripheral quantitative computed tomography to quantify right femoral and tibial J and soft tissue cross‐sectional areas, and force plate mechanography to quantify peak power output and maximum force of the right limb, among athletic women and control subjects.

**Results:**

Lower limb bone J exhibited strong relationships with estimated force but not power between both groups. Among controls, the strongest relationships between force and J were found at the midshaft femur. Among athletes, these relationships shifted to the tibia, regardless of body size, likely reflecting functional strain related to the major knee extensors and ankle plantarflexors. Together, muscle force and stature explained as much as 82 and 48% of the variance in lower limb bone J among controls and athletes, respectively.

**Discussion:**

Results highlight the importance of considering relevant muscle function variables (e.g., force and lever arm lengths) when interpreting behavioral signatures from skeletal remains. Future work to improve the estimation of muscle force from skeletal remains, and incorporate it with lever arm length into analyses, is warranted. Results also suggest that, in doing so, functional relationships between a given section location and musculature should be considered.

## 1 INTRODUCTION

Biological anthropologists often utilize variation in limb bone diaphyseal cross‐sectional geometry (CSG) to infer patterns of loading in the past related to locomotion, mobility, and/or habitual behavior. The polar second moment of area (J) provides a measure of cross‐sectional bending and torsional rigidity and is often used in behavioral interpretations, as it is readily quantifiable from lower limb diaphyses in skeletal remains, can be estimated accurately from much of the periosteal contour alone (Macintosh, Davies, Ryan, Shaw, & Stock, 2013; Stock & Shaw, 2007), and varies substantially in relation to both inferred behavior (Marchi, 2008; Ruff et al., 2015; Ruff, Larsen, & Hayes, 1984; Stock & Pfeiffer, 2001) and known behavior (Macintosh, Pinhasi, & Stock, 2017; Macintosh & Stock, 2019; Niinimäki et al., 2019; Shaw & Stock, 2009a; Shaw & Stock, 2009b). Observed differences in lower limb bone J relative to known and inferred mobility derive from the responsiveness of bone surfaces to prevailing strain conditions (Garn, 1972; Gosman, Stout, & Larsen, 2011). Known as functional adaptation (Ruff, Holt, & Trinkaus, 2006), this process involves bone deposition and resorption relative to localized strain (Ruff, 2008; Seeman, 2008) and can alter the cross‐sectional distribution of bone tissue. The most mechanically relevant forces acting on the lower limbs, bending and torsion (Ruff & Hayes, 1983), produce stresses about a neutral cross‐sectional axis (bending) or centroid (torsion), whose magnitudes are zero at these neutral locations and increase proportionally as distance from them increases (Frankel & Nordin, 1980; Ruff & Hayes, 1983). This means that the addition of bone to the periosteal surface maximizes diaphyseal J, as it is bone fibers in the periosteal region that must resist the majority of stresses during loading (Jepsen, 2009). By quantifying variation in limb bone diaphyseal J within and among populations in the past, bioarchaeological analyses can infer relative amounts of bending/torsion during life based on the bone's functional response, particularly at the periosteal surface.

### 1.1 Behavioral signals in lower limb bone cross‐sectional geometry

Functional adaptation is influenced by a variety of loading‐related parameters such as weight‐bearing, body size and shape, ground reaction forces, muscle contractions, and the magnitude and frequency of loading (Niinimäki et al., 2019; Robling, 2009). Loading also modulates the normal sequence of bone deposition and resorption that is ultimately under biochemical regulation, involving reproductive hormones, growth hormones, and others (Gosman et al., 2011; Libanati, Baylink, Lois‐Wenzel, Srinvasan, & Mohan, 1999; Parfitt, 2002; Tanner, 1989). This means that variation in hormonal status, as well as in the dietary‐derived nutrients required for normal hormone secretion (Garn, Guzmán, & Wagner, 1969; Garn, Rohmann, Béhar, Viteri, & Guzmán, 1964; Kenney, McCoy, & Williams, 1994), can alter cross‐sectional bone distribution in ways unrelated to loading. Because many of these factors are simply unknown among past individuals, inferences about behavior based on bioarchaeological analyses of skeletal variation tend to remain quite broad. When inferred behavior differs distinctly, for example among foragers relative to farmers or industrialized populations, the former tend to exhibit higher size‐standardized mean midshaft femoral and tibial J, attributed to relatively higher terrestrial mobility (Bridges, Blitz, & Solano, 2000; Ruff et al., 1984). These inferences are supported by consistent and similar differences among living athletes relative to controls, where behavior is known (Macintosh & Stock, 2019; Niinimäki et al., 2019; Shaw & Stock, 2009a; Shaw & Stock, 2009b). However, our ability to infer more nuanced detail about the loading environment that has shaped patterns in limb bone structural variation in the past is limited by an inability to reconstruct more specifically the parameters exerting force on the bones during locomotor behaviors.

One of these parameters is muscular contraction, which accounts for a substantial amount of the force applied to bone in normal daily movement (Burr, 1997; Robling, 2009). So closely related are muscle and bone that the biomechanical relationship between the two is typically viewed as a functional “muscle‐bone” unit (Fricke & Schoenau, 2007; Schoenau & Frost, 2002). As the lower limb impacts the ground and pushes back off of it during locomotor movements, ground reaction forces (GRFs) are exerted on the limb bones, reflecting the impact loading produced by accelerating and decelerating the body as it contacts the ground (Vainionpää et al., 2007). More specifically, GRFs reflect the activity of the skeletal muscles as they contract eccentrically during the braking phase to slow the body's dropping center of mass and contract concentrically to raise it again during the push‐off phase (Ishikawa & Komi, 2008). As a result, variation in activities performed during life can induce substantial variation in the loading to which bones must functionally adapt.

The performance of particularly high‐impact activities can exert substantially higher GRFs on the lower limb bones during ground contact than is typical of normal daily movement. For example, the loading associated with some sports, such as triple jump, can exert peak impact GRFs of over 15x body weight (Perttunen, Kyröläinen, Komi, & Heinonen, 2000). These high impacts have been associated with substantial bone functional adaptation in the lower limb, leading to increases of over 20% in midshaft tibial bone strength indices (BSIs) among triple jump athletes relative to controls (Heinonen, Sievänen, Kyröläinen, Perttunen, & Kannus, 2001). In contrast, sport‐specific loading regimes involving lower‐impact activities, for example power‐lifting or rowing, may not necessarily provide sufficient loading stimulus to enhance limb bone CSG properties among athletes relative to controls, despite substantial muscular effort (Macintosh & Stock, 2019; Niinimäki et al., 2017, 2019; Nikander, Sievänen, Uusi‐Rasi, Heinonen, & Kannus, 2006). In some instances, muscle activity explains *more* variance in bone strength parameters than sport‐related impact loading. For example, among 133 premenopausal female national‐level athletes and controls, estimated muscle performance‐related joint moments explained 42% more of the variance in BSI at the tibial midshaft than did sport‐related impact loading modality (Nikander et al., 2006). This is supported by Schipilow, Macdonald, Liphardt, Kan, and Boyd (2013), who demonstrated that muscle strength accounted for 13% *more* of the variance in distal tibial failure load (N), as quantified from finite element analysis, than did sport‐related impact loading relative to controls. In the proximal femur, when the effect of gluteal muscle size, strength, and body size are removed, sport‐related ground impact does still exert a significant effect at midshaft among females, but only in the presence of very high ground impacts performed at a high rate (Niinimäki et al., 2019). Given this complexity, interpreting the loading environment experienced by limb bones during life from skeletal remains alone can be challenging, and would benefit from the development of more rigorous methods of estimating muscle activity in the past.

One means of estimating local muscle force among living humans is by quantifying muscle cross‐sectional areas (MCSAs), as these are roughly proportional to the force that muscle can produce among both males and females (Maughan, Watson, & Weir, 1983b). MCSAs have demonstrated significant explanatory power for cross‐sectional geometry, bone areas, and bone strength throughout the lifespan (Heinonen, Mckay, Whittall, Forster, & Khan, 2001; Macdonald, Kontulainen, Petit, Janssen, & McKay, 2006; Rantalainen, Nikander, Kukuljan, & Daly, 2013). However, the estimation of MCSAs from skeletal remains alone has proven difficult, and existing attempts to do so have had mixed results. Though Slizewski, Onau, Shaw, and Harvati (2013) found that radial cortical bone area predicted forearm MCSA among living humans with percent *SE*s of between ~11 and 15%, accuracy varied highly by age and sex. Among young males, Shaw (2010) found that muscle areas were generally poor predictors of tibial cross‐sectional bone area and J, with relationships largely reflecting covariation with body size.

These studies assessed relationships between MCSAs and the immediately adjacent cortical bone obtained from the same section location through the limb. However, cortical bone parameters may be more influenced by functionally‐related MCSAs located elsewhere in the limb, rather than immediately adjacent to the cross‐section of interest. For example, tibial midshaft BSI is more strongly associated with muscle area in the thigh than the calf among older men (>65 years of age) (Rantalainen et al., 2013), while the relative influence of gluteal muscle loading on proximal femoral geometry has also been shown to vary by cross‐sectional location (Niinimäki et al., 2019). As a result, the estimation of muscle from bone in the past may prove more successful if we can better account for functional muscle‐bone relationships within the limb. An improved understanding of these relationships may also help explain some of the regional variation in CSG properties within the lower limb that is consistently documented among archaeological and living populations. Tibial diaphyseal structural properties often appear to reflect inferred loading more clearly than femoral diaphyseal properties (Davies & Stock, 2014; Macintosh, Pinhasi, & Stock, 2014; Macintosh & Stock, 2019; Stock, 2006), which is currently attributed to a wider range of influences, such as body size and breadth, acting on the femoral diaphysis (Davies & Stock, 2014). However, the contribution of functional relationships between tibial CSG and the powerful knee extensors of the *proximal* limb segment could hold explanatory power for these regional patterns in the limb, at least in part.

Though a muscle's cross‐sectional area is a good proxy for its ability to produce force, muscle strength per unit of cross‐sectional area exhibits considerable variation among subjects, attributable to variation in fiber type composition (Kanehisa, Ikegawa, & Fukunaga, 1994; Maughan et al., 1983b; Maughan, Watson, & Weir, 1983a). Thus, more direct measures of functional strength may be more accurate. Vertical GRFs can be measured directly from subjects while jumping on a force plate, and these GRFs can be used to estimate maximum muscle force and power produced during whole‐limb movements (Anliker, Rawer, Boutellier, & Toigo, 2011; Binkley & Specker, 2008; Rantalainen, Heinonen, Komi, & Linnamo, 2008). The highest peak voluntary GRFs on the force plate are produced by single‐leg hopping (Veilleux & Rauch, 2010), providing a good estimate of maximum voluntary muscle force (Fmax) of the lower limb ankle plantarflexors (Anliker et al., 2011). Lower limb Fmax derived from single‐leg hopping has demonstrated stronger relationships with tibial bone mass than does calf MCSA in both sexes (Anliker et al., 2011), and has been associated with increased periosteal expansion at the midshaft tibia (Hardcastle et al., 2014). As maximum and polar second moments of area are maximized by periosteal expansion (Ruff, 2008; Ruff & Hayes, 1983), we might expect them to exhibit a strong relationship with force plate‐derived Fmax. In support of this, maximum second moments of area (Imax; mm^2^) at the midshaft tibia have demonstrated significant positive correlations with maximum voluntary force among young adult women (Rantalainen et al., 2008).

Mechanography also allows for the estimation of peak power output (Pmax; W), derived from force estimates combined with velocity during the upward phase of a bilateral countermovement jump. This peak power output is thought to reflect primarily the actions of the hip and knee extensors in addition to those of the ankle plantarflexors (Hardcastle et al., 2014), and Pmax has shown significant associations with tibial compressive and bending strength indices among both sexes (Binkley & Specker, 2008; Hardcastle et al., 2014; Rantalainen et al., 2010). However, of relevance to maximizing polar second moments of area, the relationship between Pmax and cortical bone strength may be reflecting reduced endosteal resorption rather than enhanced periosteal expansion (Hardcastle et al., 2014). This suggests that Pmax may not exhibit a strong relationship with J specifically, though this remains to be determined. Currently, the extent to which limb bone CSG properties reflect whole‐limb muscle force or power among past populations is unknown.

### 1.2 The functional interaction of body size, composition, and cross‐sectional geometry

Given the close relationship between muscle and bone, bone mass and total body lean mass are strongly correlated throughout life, regardless of sex or reproductive status (Capozza, Cointry, Cure‐Ramírez, Ferretti, & Cure‐Cure, 2004; Ferretti et al., 1998). However, the importance of muscle activity in shaping lower limb bone strength parameters is such that some of the behavioral signature being inferred from skeletal variation, even among otherwise similarly‐sized individuals, may be attributable to variation in body *composition*, as well as age‐related changes in it. Body size variation is known to impact polar second moments of area through its influence on diaphyseal diameters, the amount of weight borne by the lower limb bones, the extent of mechanical stimulation on them during ground impact, the amount of contractile muscle tissue, and the lever arms about which that muscle tissue exerts bending and joint moments during movement (Niinimäki et al., 2019; Ruff, 2000; Ruff, 2018). As a result, many CSG properties scale with body mass (Parfitt, 1994; Seeman et al., 1996), and polar second moments of area must be standardized to bone length (a proxy for lever arm length) and estimated body mass prior to comparison among individuals and populations (Ruff, 2000; Ruff, 2008; Ruff, 2018). In the lower limb, this enables variation in CSG properties to be attributed more directly to functional adaptation in response to behavior while controlling for the influence of variation in body size (Ruff, 2000).

However, controlling for the effect of lever arm length as a component of body size standardization makes it difficult to explore the contribution of muscle activity to the behavioral signal that we are detecting from skeletal remains. Our potential to explain variation in midshaft diaphyseal bone strength parameters would be improved if we could better estimate muscle force from bone, and incorporate force estimates alongside bone length into statistical analyses. For example, estimated joint moments alone (proxied from stature and maximum muscle force) explain almost 50% of the variance in midshaft tibial BSI among young female athletes and controls, and 42% *more* variance than ground impact loading (Nikander et al., 2006). Further, maximum torques directly measured during knee extension are highly correlated with MCSA of the active muscle group multiplied by stature as a proxy for lever arm length among both males and females (Schantz, Randall‐Fox, Hutchison, Tydén, & Astrand, 1983). At present, we do not know how much of the variation in lower limb J, within or between limbs, is explained by muscle activity or by variation in relative lean mass. To do so requires the development of better methods of estimating, and then incorporating, relevant muscle function variables (e.g., force and lever arm lengths) into our statistical comparisons. The first step to doing so is building a more accurate picture of the relationships between muscle size, function, and commonly used CSG properties and section locations throughout the weight‐bearing lower limb as a whole.

### 1.3 Objectives

In the current study, we seek to determine what relationship estimated lower limb muscle force and power (proxied from thigh and calf MCSA and whole‐limb force plate mechanography) have with polar second moments of area quantified at commonly used section locations in bioarchaeology: the midshaft femur, proximal tibia, and midshaft tibia. We assess these relationships among a group of female athletes and control subjects from whom several key relationships have already been demonstrated. First, significant enhancement of midshaft femoral and tibial J relative to controls was only documented among sports involving ground impact loading (soccer and endurance running); high muscle magnitudes alone (rowing) were not sufficient to elicit significant change in midshaft lower limb bone J relative to controls (Macintosh & Stock, 2019). Second, midshaft femoral and tibial J were most strongly correlated with total body lean mass (*r* ≤ .72), followed by body mass (*r* ≤ .60), and fat mass (*r* ≤ .27), as quantified from bioelectrical impedance (Pomeroy, Macintosh, Wells, Cole, & Stock, 2018). Third, midshaft tibial J was a stronger predictor of total body lean mass than of body mass (adjusted *R* ^2^ of 0.51 vs. 0.36, respectively), particularly when bone length was included in regression models (adjusted *R* ^2^ of 0.6 vs. 0.42, respectively) (Pomeroy et al., 2018).

Among this mixed sample of females, we assess relationships between muscle size, function, and cross‐sectional J among two groups split by physical activity history: recreationally‐active control subjects with no history of sport‐related impact loading or intensive muscular activity, and active athletes with significant current and past histories of competitive sport. Among the athlete group, sport‐related loading characteristics varied (see Section 2) but all were combined into one group in order to more accurately reflect the variable behaviors and activity histories of individuals from fossil, prehistoric, or archaeological populations. Our three main research questions, and associated hypotheses, are as follows:

1. To what extent do estimates of regional and/or whole‐limb muscle force and power influence femoral and tibial polar second moments of area?
  We hypothesize that estimates of maximum force and peak power output will be significantly predictive of polar second moments of area at all section locations, but that peak power output may be less strongly related to lower limb bone J than maximum force.
2. How does the relationship between J and MCSAs pattern regionally within the lower limb?
  Reflecting functional relationships, we hypothesize that proximal and midshaft tibial J will be more strongly correlated with thigh MCSA than with calf MCSA and that midshaft femoral J will be more strongly correlated with thigh MCSA than with calf MCSA. We further hypothesize that overall relationships between femoral J and MCSAs may be weaker than is the case with tibial J, due to our inability to account for the influence of functionally‐related gluteal musculature on the midshaft femur.
3. Are estimated force (MCSAs and Fmax) and/or power variables, in combination with a proxy for lever arm length (stature), better independent predictors of variance in femoral and/or tibial J than body mass and stature alone?
  We hypothesize that regression models including a proxied measure of muscle force or power will explain more variance in J than models incorporating only stature and body mass.

## 2 METHODS

### 2.1 Participants

A total of 102 women took part in the study, and all provided written informed consent prior to participation. All participants were healthy, pre‐menopausal women aged 19–34 years, primarily of European descent. They included a range of current and past physical activity levels, from recreationally‐active women through to women with elite sporting histories. Participants had neither recent soft tissue injuries that affected muscle performance nor medical conditions or medication use known to affect bone metabolism. All participants completed a questionnaire assessing their past and current recreational and competitive physical activity histories, and a variety of nutritional and hormonal factors known to affect bone, including age at menarche, family history of osteoporosis, active avoidance of dairy products, past and current estrogen‐based hormonal contraceptive use, age at first use of estrogen‐based hormonal contraceptives, and any history of menstrual irregularity, defined as any history of amenorrhea or oligomenorrhea (fewer than 10 periods in 12 months) not clearly related to contraceptive use (e.g., intrauterine devices).

The control subjects (*N* = 34) had never taken part in competitive sport and had no current or past participation of more than 3 hr per week of intensive weight‐bearing sport, but some were active at a recreational level in yoga, cycling, pilates, swimming, and other non‐weight‐bearing exercise. The athlete group (*N* = 68) consisted of women with current and previous participation in sport as high as national‐team level, often participating at a competitive level in multiple different sports throughout their lives. Athletes were recruited via their current participation in one of three sports (rowing, endurance running, soccer), but for the purposes of this study were not screened by previous or concurrent loading history, so represent women with a range of impact loading histories. The sports most frequently reported in addition to rowing, endurance running (middle‐distance through ultramarathon) and soccer were: triathlon, swimming, field hockey, rugby, cricket, netball, basketball, volleyball, tennis, lacrosse, gymnastics, and equestrian. No athlete had any significant injury within the past year that rendered them inactive for over 1 month. The research was approved by the Cambridge University Human Biology Research Ethics Board (HBREC.2015.25 and HBREC.2016.14), and the NHS Health Research Authority NRES Committee East of England—Cambridge East (15/EE/0017).

### 2.2 Anthropometry

Stature was measured to the nearest 0.01 cm using a SECA 274 stadiometer, and was used as a proxy for lever arm length for the purposes of this study. Body mass was recorded to the nearest 0.1 kg with a SECA electronic scale. Femoral length and maximum tibial length were obtained from participants using sliding calipers according to the methods in International Standards for Anthropometric Assessment (International Society for the Advancement of Kinanthropometry, 2001). For the femur, maximum length was measured as the distance between the proximal border of the greater trochanter and the distal margin of the lateral condyle, and does not reflect the true maximum length of the bone. For the tibia, maximum length was measured as the distance between the proximal medial border of the tibial plateau and the distal margin of the medial malleolus.

### 2.3 Bone, muscle, and fat cross‐sectional parameters

**FIGURE 1:**
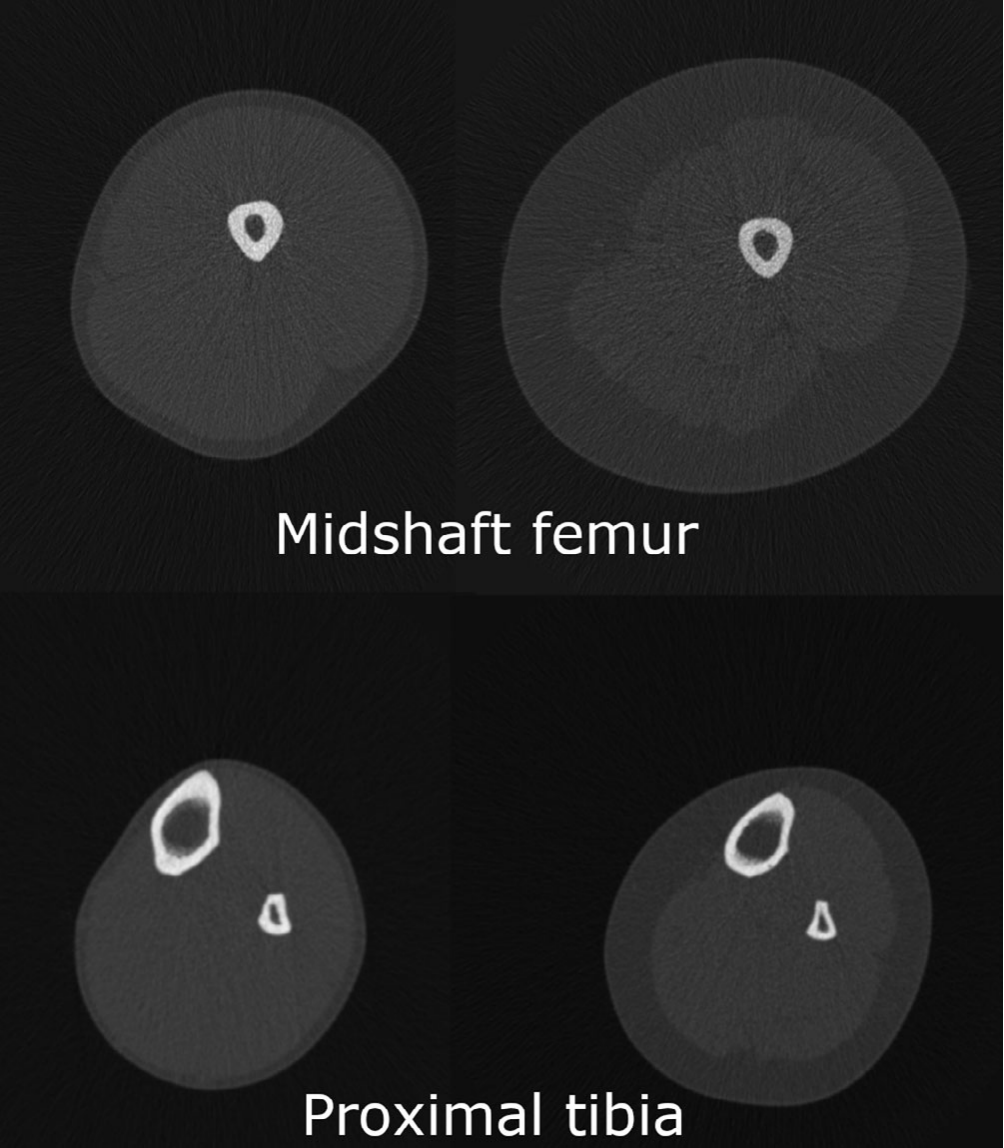
pQCT slices at the two section locations from which soft tissue parameters were quantified, demonstrating the range of cross‐sectional variation in muscle and subcutaneous fat in the sample

All cross‐sectional bone images were collected using peripheral quantitative computed tomography (pQCT; XCT‐3000; Stratec Medizintechnik GmbH, Pforzheim, Germany) at the University of Cambridge. Cross‐sections were obtained from the midshaft femur and tibia, at 50% of maximum length from the distal end, and from the proximal tibia, at 66% of maximum length from the distal end (see Figure 1). However, because it was not possible to obtain true maximum length from the femur among living participants, the “midshaft” femoral slice used here is slightly distal to true midshaft, though is still taken close to diaphyseal transverse cross‐sectional minima. Muscle cross‐sectional areas were used as proxies for localized muscle force that are not reliant on participant skill or motivation (Frank, Lorbergs, Chilibeck, Farthing, & Kontulainen, 2010). Both muscle and subcutaneous fat cross‐sectional areas (MCSA and FCSA; mm^2^) were quantified at the midshaft femur (thigh MCSA and FCSA) and proximal tibial scans (calf MCSA and FCSA), using Macro analyses in the pQCT manufacturer software (XCT, version 6.2.0), with Contour Mode 1 (−100 mg/cm^3^), Peel Mode 2 (40 mg/cm^3^), Cort mode 1 (710 mg/cm^3^), and an F03F05F05 muscle filter applied. Cross‐sectional pQCT images were then imported into ImageJ (http://rsbweb.nih.gov/ij/). The polar second moment of area (J; mm^4^), a measure of twice average bending and torsional rigidity, was quantified using the bone image analysis plug‐in BoneJ (Doube et al., 2010), with the “Optimize Threshold” function used to isolate cortical bone from surrounding tissue.

### 2.4 Whole‐limb performance parameters

Whole lower limb maximum force and power were evaluated using a Leonardo Mechanograph Ground Reaction Force Platform (NOVOTEC Medical GmbH, Pforzheim, Germany) and manufacturer's software (Leonardo Mechanography version 4.4). This force plate is split into two sections that enable measurement of the left and right legs separately. The Leonardo Mechanograph system measures force applied to the plate over time, but also measures acceleration, allowing it to quantify vertical velocity and ultimately power output from these force and velocity measurements (Dionyssiotis, Galanos, Michas, Trovas, & Lyritis, 2009). The force platform was calibrated prior to each assessment, and subjects performed all jumps in socked or bare feet.

**FIGURE 2:**
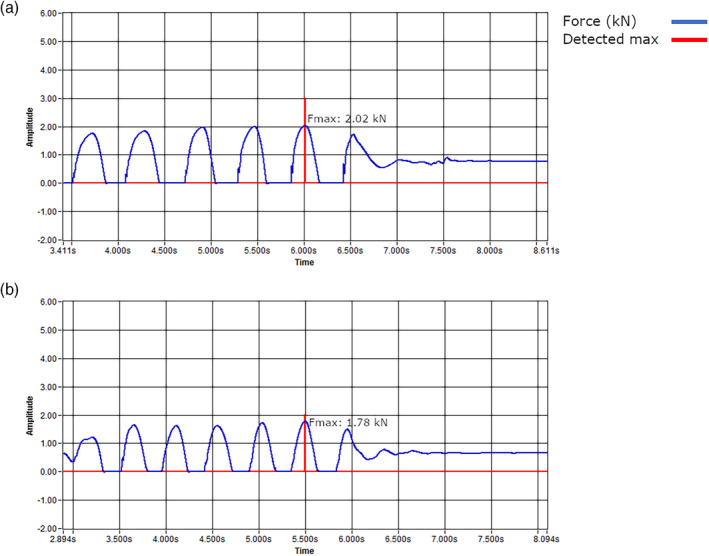
Ground reaction force curves during multiple single‐leg hopping (M1LH) of an (a) athlete and (b) control subject, each most representative of their group's mean Fmax. Detected Maximum tracing (in red) is indicative solely of the timing of peak force and does not reflect force amplitude

Maximum voluntary force (Fmax; kN) of the right leg was obtained from the lift‐off phase during multiple single‐leg hopping (M1LH) on the ball of the foot. Force was acquired from single‐leg hopping because this movement pattern produces the highest peak GRFs on the force plate (Veilleux & Rauch, 2010), so is the best proxy for maximum voluntary force exerted by the ankle plantarflexors (Anliker et al., 2011). Ground reaction force curves during multiple single‐leg hopping of an athlete and control subject, each most representative of their group's mean Fmax, are presented in Figure 2. The multiple single‐leg hopping procedure begins with the subject in an upright standing position, after which they lift the non‐testing leg off the plate, and begin hopping on the testing leg while keeping the knee stiff. Subjects were instructed to stand still on both legs until hearing a beep from the computer, at which point they were to perform six to eight consecutive single‐leg hops on the ball of the foot, keeping their heel off of the plate at all times, with the objective of minimizing contact time with the plate. Upon completion of each set of hops, the subjects were asked to stand still on both legs again, until hearing two beeps, indicating completion of the test. Each subject was then allowed to warm up and perform approximately 10–15 submaximal hops to practice. For testing, each subject performed three sets of six to ten consecutive single‐leg hops on the ball of the foot. Any hop in which the heel contacted the force plate was excluded; of remaining hops, the one producing the highest Fmax relative to their body weight (indicated as the detected maximum in Figure 2) was identified, and the raw Fmax used for analyses.

**FIGURE 3:**
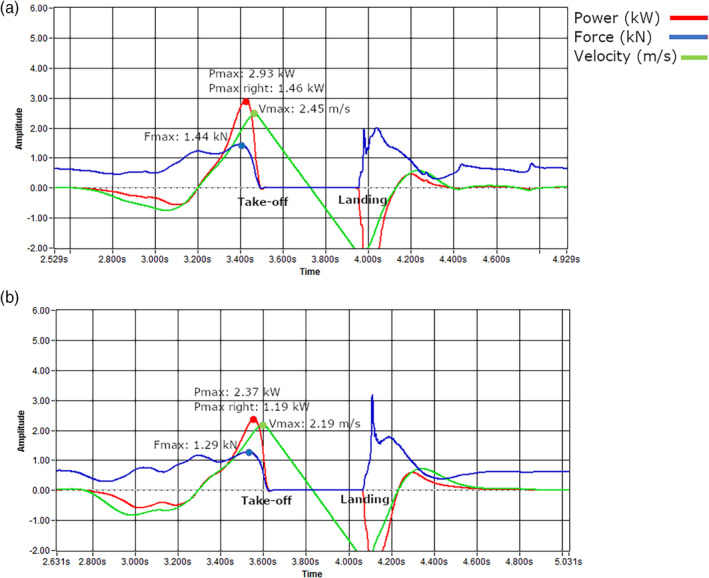
Power, force, and velocity curves during the countermovement jump (S2LJ) of an (a) athlete and (b) control subject, each most representative of their group's mean Pmax. Each subject's maximum force and velocity are also provided. Units for the *y*‐axis are as follows: power, kW; force, kN; velocity, m/s. Each subject's take‐off and landing point are indicated; all Pmax values were obtained during the upward phase of the jump, just prior to take‐off. Maximum power during take‐off was obtained at 3.43 s in (a), and 3.55 s in (b)

Peak power output (Pmax; kW) was obtained for the right leg during the lift‐off phase of a single two‐legged countermovement jump (S1LJ). The countermovement jump provides insight into an individual's neuromuscular capabilities while remaining resistant to participant fatigue and requiring minimal habituation prior to testing (Cormie, McBride, & McCaulley, 2009; McMahon, Suchomel, Lake, & Comfort, 2018). Power, force, and velocity curves during the countermovement jump of an athlete and control subject, each most representative of their group's mean Pmax, are presented in Figure 3. The jump begins with the subject in an upright standing position, followed by an initial lowering of the center of mass as the subject squats (the countermovement), during which energy is stored in their elastic soft tissues, and then the immediate driving of the body vertically off of the force plate (Dionyssiotis et al., 2009; McMahon et al., 2018). Subjects were instructed to stand as still as possible on the plate until hearing a beep from the computer, at which point they were to rapidly squat to their preferred depth and immediately jump vertically, as high and fast as they could, using both legs as well as freely swinging their arms to elevate their head and chest as high as possible above the ground. Each participant was also asked to soften their landing by absorbing some of the impact through flexing their hips, knees, and ankles, and then to stand upright and still until hearing two beeps, signifying completion of that attempt. Each participant was allowed to warm up and familiarize herself with the force plate prior to testing, after which each subject performed three attempts. The jump in which the subject obtained the highest total peak power output (produced by both legs together) relative to their body weight during the lift‐off phase was identified, and raw Pmax from the right leg specifically was isolated from the right half of the force plate and used for analyses.

### 2.5 Statistical analyses

All data were tested for normality by assessing standardized skewness (skew/*SE*), with values between −3.29 and 3.29 considered normally distributed. Variables that were not normally distributed were log10 transformed prior to analyses. Independent samples *t* tests were used to assess differences between athletes and control subjects in 14 continuous parameters. Chi‐squared or Fisher's Exact tests were used to compare five categorical parameters. Bivariate correlations were used to assess the relationships between limb bone polar second moments of area at each section location and stature, body mass, muscle areas, and performance variables, and partial correlations were used to do so after controlling for stature and body mass. Bivariate correlations were also used to assess the relationship between muscle areas and body size parameters. Age did not significantly correlate with any muscle or bone variables in our sample of participants, so was not included in regression models.

Hierarchical linear regression models were constructed to assess the variance in muscle area (MCSA) and whole‐limb performance variables (Pmax, Fmax) accounted for in J at different locations in the limb. Hierarchical models for J at the midshaft femur, proximal tibia, and midshaft tibia were constructed, and each was adjusted for baseline stature and body mass. Subsequently, four models were built for each section location, with either thigh MCSA, calf MCSA, Pmax, or Fmax entered. For each model, multicollinearity was assessed based on tolerance, variance inflation factors, and variance proportions; no regression models violated the assumptions of multicollinearity. This procedure was performed separately among athletes and control subjects. Statistical analyses were performed using SPSS Version 24, with significance set at *p* < .05.

## 3 RESULTS

Descriptive statistics for all variables, and results of independent samples *t* tests between athletes and controls, are provided in Table 1. The athlete group was, on average, significantly taller (*p* = .018) than the control group and had significantly larger absolute thigh and calf MCSA (all *p* < .001) but significantly smaller absolute thigh and calf FCSA (*p* = .001). Despite these differences in body composition, there were no significant differences in body mass between the two groups. Thus, our two groups were similar in body mass but differed significantly in the proportion of that mass that is capable of generating performance‐related bending and joint moments on limb bone diaphyses. Images of pQCT slices at the midshaft femur and proximal tibia demonstrating the range of variation in soft tissue parameters in the sample are provided in Figure 1. Given their substantially larger stature and absolute muscle areas, athletes were able to generate significantly higher raw Fmax and Pmax (*p* < .001) than controls. Athletes also had significantly higher raw values of J throughout the limb (all *p* < .001). There were no significant differences between the athlete and control group in any questionnaire‐based self‐reported dietary or hormonal parameters.

**TABLE 1:** Descriptive statistics (*N* = 102)

	Athletes (*N* = 68)		Controls (*N* = 34)	
Variable	Mean (*SD*)	Range	Mean (*SD*)	Range
Age (yr)	*23.93 (4.28)*	19–34	*23.47 (3.54)*	19–32
Stature (cm)	171.14 (7.39)*	152.90–188.20	167.44 (7.53)	154.20–183.40
Body mass (kg)	*65.00 (8.71)*	50.70–86.00	*61.79 (11.16)*	40.00–92.10
Thigh MCSA (mm^2^)	*13*,*480.85 (1*,*586.50)* *	10,118.50–17,336.25	*10*,*686.41 (1*,*711.67)*	7,824.50–16,799.75
Thigh FCSA (mm^2^)	*6*,*644.51 (2*,*472.40)* *	2,408.00–15,223.50	*8*,*627.16 (3*,*431.19)*	4,037.25–18,880.75
Calf MCSA (mm^2^)	*6*,*885.85 (782.00)* *	5,468.75–9,270.75	6,166.18 (1,021.27)	4,663.00–8,401.50
Calf FCSA (mm^2^)	*2*,*251.73 (722.90)* *	768.25–4,934.50	*2*,*925.88 (903.91)*	1,688.00–5,036.00
Pmax (kW)	1.47 (0.26)*	0.83–2.32	1.19 (0.25)	0.74–1.75
Fmax (kN)	*2.03 (0.28)* *	1.47–2.95	1.76 (0.31)	1.15–2.41
Polar second moment of area (J; mm^4^)				
Femur 50%	*44*,*254.45 (9*,*371.87)* *	24,115.20–72,081.30	*34*,*505.07 (8*,*683.38)*	20,543.70–62,098.02
Tibia 66%	*48*,*535.74 (10*,*568.17)* *	30,949.75–76,943.29	*37*,*628.27 (10*,*220.53)*	17,826.57–73,086.96
Tibia 50%	*32*,*958.36 (7*,*113.12)* *	20,446.52–52,726.59	*25*,*775.13 (6*,*894.86)*	11,815.74–50,316.99
	Mean (*SD*) or count (% of total)	Range (where applicable)	Mean (*SD*) or count (%)	Range (where applicable)
Age at menarche (yr)	13.19 (1.39)	10–16	12.82 (1.80)	10–17
Family history of osteoporosis	7 (10.3%)	—	6 (17.6%)	—
Dairy avoidance	7 (10.3%)	—	4 (11.8%)	—
Past hormonal contraceptive use	49 (72.1%)	—	26 (76.5%)	—
Current hormonal contraceptive use	27 (39.7%)	—	17 (50.0%)	—
Age at first use (yr)	17.59 (2.46)	12–24	17.46 (3.19)	10–24
History of menstrual irregularity	23 (33.8%)	—	12 (35.3%)	—

*Note:* All muscle and bone variables derived from the right limb only; italicized variables were log10 transformed prior to analyses.

* Statistically significant difference between athletes and controls.

### 3.1 Body size

**FIGURE 4:**
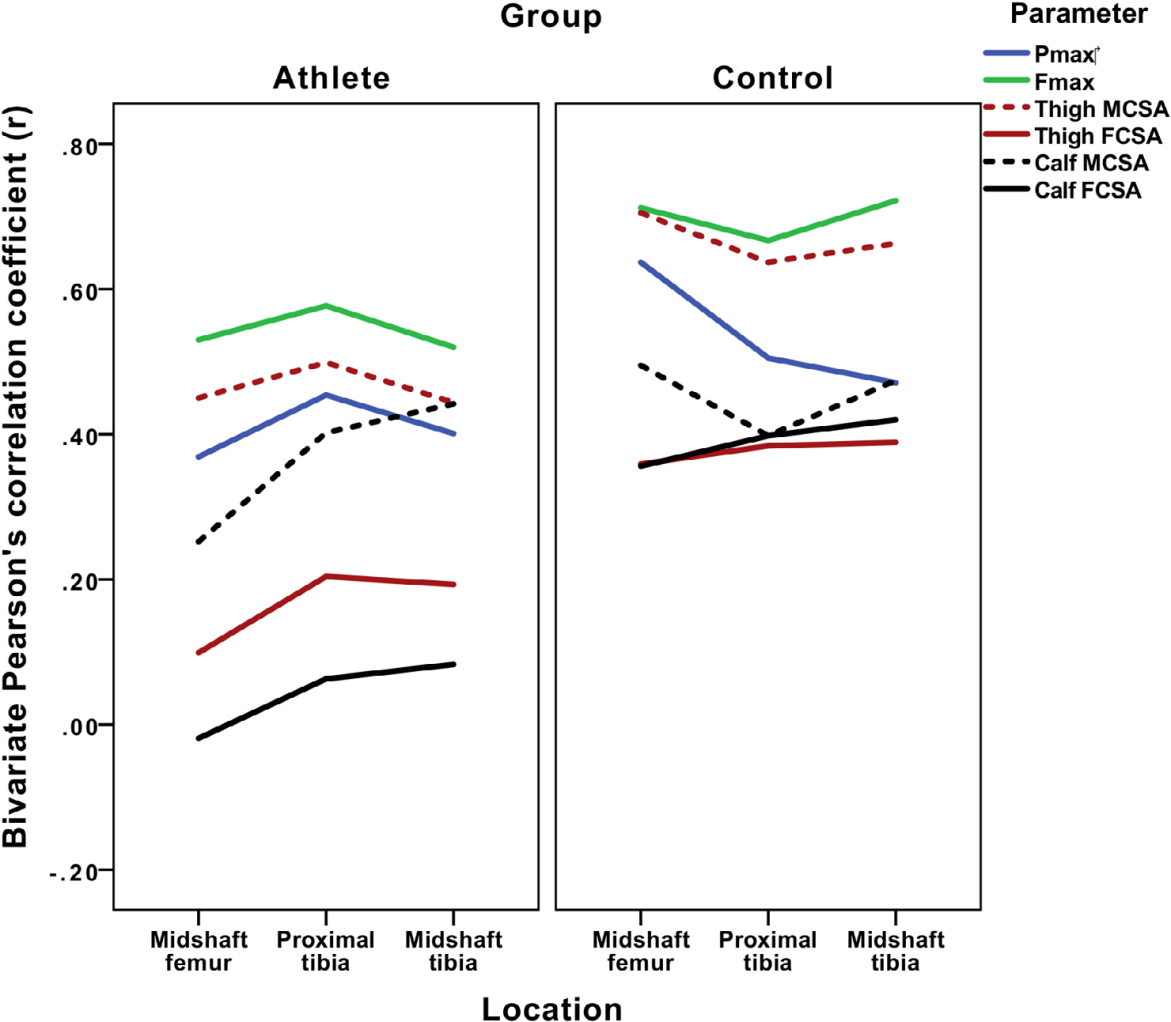
Bivariate correlations between regional polar second moments of area (J) and both regional soft tissue areas and dynamic performance variables

The results of bivariate correlations are provided in Table 2 and depicted in Figure 4. Limb bone J at all section locations was significantly correlated with stature and body mass, but the extent of the relationship and its patterning throughout the limb differed between athletes and controls. Among control subjects, stature and body mass were more highly correlated with lower limb J (*r* = .572–.754) than among athletes (*r* = .524–.580), and these relationships were strongest at midshaft locations, particularly in the femur (stature: *r* = .754; body mass: *r* = .648; all *p* < .001), and weakest at the proximal tibia (stature: *r* = .629; body mass: *r* = .612; all *p* < .001). In contrast, among athletes, relationships between body size variables and limb bone J were consistently *strongest* at the proximal tibia (stature: *r* = .580; body mass: *r* = .575; all *p* < .001), and weaker at the midshaft locations.

**TABLE 2:** Bivariate and partial correlations assessing relationships between regional lower limb bone J and both regional soft tissue areas and dynamic performance variables (*N* = 102)

	Polar second moment of area (J; mm^4^)					
	Athletes (*N* = 68)			Controls (*N* = 34)		
	Femur 50%	Tibia 66%	Tibia 50%	Femur 50%	Tibia 66%	Tibia 50%
Age (yr)	0.025	0.033	0.107	0.215	0.102	0.033
Stature (cm)	**0.546**	**0.580**	**0.538**	**0.754**	**0.629**	**0.572**
Body mass (kg)	**0.521**	**0.575**	**0.524**	**0.658**	**0.612**	**0.635**
Pmax (kW)	**0.369**	**0.454**	**0.401**	**0.637**	**0.505**	**0.471**
Fmax (kN)	**0.530**	**0.577**	**0.520**	**0.712**	**0.667**	**0.722**
Thigh MCSA (mm^2^)	**0.450**	**0.499**	**0.444**	**0.705**	**0.637**	**0.663**
Thigh FCSA (mm^2^)	0.099	0.204	0.193	**0.359** ^a^	**0.384** ^a^	**0.389** ^a^
Calf MCSA (mm^2^)	**0.254** ^a^	**0.402**	**0.442**	**0.495**	**0.398** ^a^	**0.474**
Calf FCSA (mm^2^)	−0.019	0.063	0.083	**0.356** ^a^	**0.398** ^a^	**0.420** ^a^
*Controlling for stature and body mass*						
Pmax (kW)	−0.022	0.061	0.030	0.203	−0.012	−0.093
Fmax (kN)	**0.338**	**0.372**	**0.316** ^a^	**0.579**	**0.435** ^a^	**0.523**
Thigh MCSA (mm^2^)	0.207	0.238	0.191	**0.570**	**0.368** ^a^	**0.374** ^a^
Thigh FCSA (mm^2^)	**−0.404**	**−0.278** ^a^	−0.213	−0.148	−0.017	−0.136
Calf MCSA (mm^2^)	0.175	**0.363**	**0.423**	0.259	0.074	0.183
Calf FCSA (mm^2^)	**−0.486**	**−0.427**	**−0.321**	−0.277	−0.045	−0.046

*Note:* Bold indicates statistical significance.

a Significance at *p* < .05; all other bolded correlations significant at *p* < .01.

### 3.2 Soft tissue areas

The extent of the relationship between body size variables, MCSA, and FCSA is assessed in Table 3. The body mass of control subjects was more highly correlated with their lower limb subcutaneous fat areas (thigh FCSA: *r* = .843; calf FCSA: *r* = .734; all *p* < .01) than their muscle areas (thigh MCSA: *r* = .668; calf MCSA: *r* = .361; all *p* < .01). In contrast, the body mass of athletes was more highly correlated with their lower limb muscle areas (thigh MCSA: *r* = .812; calf MCSA: *r* = .591; all *p* < .01) than their fat areas (thigh FCSA: *r* = .733; calf FCSA: *r* = .643; all *p* < .01). Stature exhibited weaker relationships with soft tissue areas than did body mass, but exhibited a significant positive correlation with mean thigh MCSA (*r* = .406, *p* < .01) among controls, and with mean calf MCSA (*r* = .251, *p* < .05) among athletes.

**TABLE 3:** Pearson's correlations assessing relationships between regional MCSAs and body size variables

	Thigh MCSA (mm^2^)	Thigh FCSA (mm^2^)	Calf MCSA (mm^2^)	Calf FCSA (mm^2^)
*Controls (N = 34)*				
Stature (cm)	**0.406**	0.056	0.080	0.222
Body mass (kg)	**0.668**	**0.843**	**0.361**	**0.734**
*Athletes (N = 68)*				
Stature (cm)	0.311	0.227	0.225	**0.251** ^a^
Body mass (kg)	**0.812**	**0.733**	**0.591**	**0.643**

*Note:* Bold indicates statistical significance.

a Significance at *p* < .05; all other bolded correlations significant at *p* < .01.

MCSAs were positively and significantly correlated with limb bone J at all section locations among both athletes and control subjects, but these relationships patterned differently within the limb (see Figure 4). Control subject MCSAs were most significantly correlated with limb bone J at the femoral midshaft, regardless of where that muscle was in the limb (thigh MCSA: *r* = .705; calf MCSA: *r* = .495; all *p* < .001). In contrast, among athletes, MCSAs were most significantly correlated with limb bone J at functionally related section locations: thigh MCSA and the proximal tibia (*r* = .499, *p* < .001), but calf MCSA and the midshaft tibia (*r* = .442, *p* < .001).

FCSAs were only significantly positively correlated with limb bone J among control subjects, where they exhibited their highest correlations with limb bone J at the tibial midshaft, regardless of where that fat was within the limb (thigh FCSA: *r* = .389; calf FCSA: *r* = .420; all *p* < .05). Athlete FCSAs did not exhibit significant bivariate correlations with limb bone J anywhere in the limb.

**FIGURE 5:**
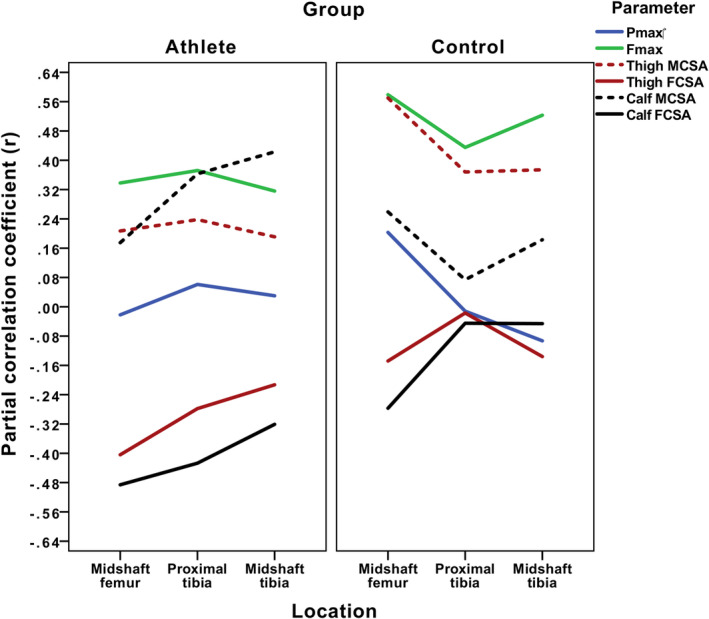
Partial correlations between regional polar second moments of area (J) and both regional soft tissue areas and dynamic performance variables

The results of partial correlations assessing relationships between muscle areas and limb bone J after controlling for stature and body mass are provided in Table 2 and depicted in Figure 5. Among controls, only thigh MCSA remained significantly positively correlated with J, with relationships highest at the midshaft femur (*r* = .570, *p* < .01) and lowest at the proximal tibia (*r* = .368, *p* < .05); FCSAs no longer exhibited any significant relationship with limb bone J. Among athletes, calf MCSA remained significantly positively correlated with J in the tibia, particularly at midshaft (*r* = .423, *p* < .01). Interestingly, controlling for stature and body resulted in significant negative correlations between thigh and calf FCSAs and J throughout the limb: at the midshaft femur (*r* = −.404 and *r* = −.486, respectively; *p* < .01), proximal tibia (*r* = −.278 and *r* = −.427, respectively; *p* < .05), and midshaft tibia (calf FCSA only; *r* = −.321, *p* < .01).

### 3.3 Whole‐limb force and power production

Raw lower limb Fmax and Pmax were significantly correlated with lower limb J among both athletes and controls, but again patterned differently. Among controls, Pmax was most highly correlated with midshaft femoral J (*r* = .637, *p* < .001) and Fmax with midshaft tibial J (*r* = .722, *p* < .001). Among athletes, both Pmax and Fmax exhibited their strongest correlations with J at the proximal tibia (Pmax: *r* = .454; Fmax: *r* = .577; all *p* < .001).

After controlling for stature and body mass, Pmax no longer exhibited any significant relationship with lower limb bone J in either group, while Fmax continued to demonstrate significant positive relationships with limb bone J at all section locations. Among controls, Fmax now exhibited its strongest relationships with midshaft femoral J (*r* = .579, *p* < .01), followed by midshaft tibial J (*r* = .523, *p* < .01) and proximal tibial J (*r* = .435, *p* < .05). Among athletes, Fmax was still most highly correlated with J at the proximal tibia (*r* = .372, *p* < .01), followed by the midshaft femur (*r* = .338, *p* < .01) and the midshaft tibia (*r* = .316, *p* < .05).

### 3.4 Explanatory power of MCSAs and whole‐limb performance variables: Control subjects

Among control subjects, hierarchical linear regression models revealed that baseline adjustment for body size (stature and body mass) explained 70.6% of the variance in midshaft femoral J (Table 4), 52.4% of the variance in proximal tibial J (Table 5), and 49.6% of the variance in midshaft tibial J (Table 6) among controls. At all three section locations, Models 3 and 4 demonstrated that thigh MCSA and Fmax explained significant portions of the variance in J, after accounting for stature. When included, these parameters each replaced body mass as an independent significant predictor explaining variance in J. The addition of Fmax explained an additional 9.2% of the variance in midshaft femoral J, 8.4% of the variance in proximal tibial J, and 12.9% of the variance in midshaft tibial J, relative to Model 1. The addition of thigh MCSA explained an additional 9.0% of the variance in midshaft femoral J, 6.0% of the variance in proximal tibial J, and 6.6% of the variance in midshaft tibial J, relative to Model 1. Calf MCSA and Pmax were never significant predictors of J at any section location among control subjects.

**TABLE 4:** Linear regression modeling investigating the explanatory power of muscle areas and dynamic performance variables for midshaft femoral J (*N* = 102)

Model	Predictors	Athletes (*N* = 68)					Controls (*N* = 34)				
		Overall *R* ^2^	Adjusted *R* ^2^	*R* ^2^ change	*β*	*p*	Overall *R* ^2^	Adjusted *R* ^2^	*R* ^2^ change	*β*	*p*
1		0.335	0.315	—			0.724	0.706	—		
	Stature (cm)				0.357	**.015**				0.586	**<.001**
	Body mass (kg)				0.269	.063				0.428	**<.001**
2		0.335	0.304	0.0			0.735	0.708	0.011		
	Stature (cm)				0.358	**.015**				0.556	**<.001**
	Body mass (kg)				0.286	.104				0.328	**.021**
	Pmax (kW)				−0.026	.860				0.157	.266
3		0.412	0.385	0.077			0.816	0.798	0.092		
	Stature (cm)				0.410	**.004**				0.600	**<.001**
	Body mass (kg)				−0.027	.875				−0.137	.424
	Fmax (kN)				0.381	**.005**				0.637	**.001**
4		0.364	0.334	0.029			0.814	0.795	0.09		
	Stature (cm)				0.386	**.008**				0.591	**<.001**
	Body mass (kg)				0.096	.582				0.009	.066
	Thigh MCSA (mm^2^)				0.229	.095				0.514	**.001**
5		0.356	0.325	0.021			0.742	0.716	0.018		
	Stature (cm)				0.412	**.007**				0.587	**.001**
	Body mass (kg)				0.173	.273				0.328	**.011**
	Calf MCSA (mm^2^)				0.159	.160				0.169	.152

*Note:* Overall *R* ^2^: coefficient of determination; adjusted *R* ^2^: adjusted coefficient of determination; *β*: standardized beta coefficient; *p*: *p*‐value. *R* ^2^ change is in overall *R* ^2^ relative to Model 1; bold values indicate significance at *p*<0.05.

**TABLE 5:** Linear regression modeling investigating the explanatory power of muscle areas and dynamic performance variables for proximal tibial J (*N* = 102)

Model	Predictors	Athletes (*N* = 68)					Controls (*N* = 34)				
		Overall *R* ^2^	Adjusted *R* ^2^	*R* ^2^ change	*β*	*p*	Overall *R* ^2^	Adjusted *R* ^2^	*R* ^2^ change	*β*	*p*
1		0.391	0.373	—			0.553	0.524	—		
	Stature (cm)				0.347	**.013**				0.460	**.001**
	Body mass (kg)				0.331	**.018**				0.431	**.002**
2		0.394	0.365	0.003			0.553	0.508	0.0		
	Stature (cm)				0.342	**.015**				0.462	**.002**
	Body mass (kg)				0.286	.09				0.439	**.018**
	Pmax (kW)				0.068	.626				−0.012	.949
3		0.476	0.452	0.085			0.637	0.601	0.084		
	Stature (cm)				0.403	**.003**				0.474	**<.001**
	Body mass (kg)				0.021	.897				−0.109	.648
	Fmax (kN)				0.400	**.002**				0.609	**.013**
4		0.426	0.399	0.035			0.613	0.575	0.06		
	Stature (cm)				0.378	**.006**				0.464	**.001**
	Body mass (kg)				0.141	.397				0.087	.669
	Thigh MCSA (mm^2^)				0.251	.054				0.422	**.038**
5		0.472	0.447	0.081			0.555	0.511	0.002		
	Stature (cm)				0.456	**.001**				0.460	**.002**
	Body mass (kg)				0.141	.325				0.394	**.02**
	Calf MCSA (mm^2^)				0.315	**.003**				0.062	.686

*Note:* Overall *R* ^2^: coefficient of determination; adjusted *R* ^2^: adjusted coefficient of determination; *β*: standardized beta coefficient; *p*: *p*‐value. *R* ^2^ change is in overall *R* ^2^ relative to Model 1; bold values indicate significance at *p*<0.05.

**TABLE 6:** Linear regression modeling investigating the explanatory power of muscle areas and dynamic performance variables for midshaft tibial J (*N* = 102)

Model	Predictors	Athletes (*N* = 68)					Controls (*N* = 34)				
		Overall *R* ^2^	Adjusted *R* ^2^	*R* ^2^ change	*β*	*p*	Overall *R* ^2^	Adjusted *R* ^2^	*R* ^2^ change	*β*	*p*
1		0.331	0.311	—			0.527	0.496	—		
	Stature (cm)				0.334	**.022**				0.382	**.008**
	Body mass (kg)				0.289	**.047**				0.485	**.001**
2		0.332	0.301	0.001			0.531	0.484	0.004		
	Stature (cm)				0.332	**.025**				0.400	**.008**
	Body mass (kg)				0.265	.132				0.546	**.005**
	Pmax (kW)				0.035	.811				−0.095	.611
3		0.398	0.370	0.067			0.656	0.622	0.129		
	Stature (cm)				0.384	**.007**				0.399	**.002**
	Body mass (kg)				0.013	.938				−0.183	.434
	Fmax (kN)				0.355	**.01**				0.753	**.002**
4		0.356	0.325	0.025			0.593	0.552	0.066		
	Stature (cm)				0.361	**.014**				0.386	**.005**
	Body mass (kg)				0.129	.464				0.125	.548
	Thigh MCSA (mm^2^)				0.211	.125				0.441	**.035**
5		0.451	0.425	0.120			0.543	0.497	0.016		
	Stature (cm)				0.468	**.001**				0.383	**.008**
	Body mass (kg)				0.056	.698				0.393	**.022**
	Calf MCSA (mm^2^)				0.384	**<.001**				0.156	.317

*Note:* Overall *R* ^2^: coefficient of determination; adjusted *R* ^2^: adjusted coefficient of determination; *β*: standardized beta coefficient; *p*: *p*‐value. *R* ^2^ change is in overall *R* ^2^ relative to Model 1; bold values indicate significance at *p*<0.05.

### 3.5 Explanatory power of MCSAs and dynamic performance variables: Athletes

Among athletes, baseline adjustment for stature and body explained only 31.5% of the variance in midshaft femoral J; in this model, body mass did not significantly explain *any* variance in J. In the tibia, both stature and body mass explained 37.3% of the variance in proximal tibial J, and 31.1% of the variance in midshaft tibial J. At the midshaft femur, Model 3 demonstrated that Fmax explained significant portions of the variance in J, after accounting for stature. The addition of Fmax explained an additional 7.7% of the variance in J, while body mass, Pmax, and thigh and calf MCSA were not significant predictors of J at the midshaft femur among athletes. At both section locations in the tibia, Models 3 and 5 demonstrated that Fmax as well as calf MCSA explained significant portions of the variance in J, after accounting for stature. When included, these parameters each replaced body mass as an independent significant predictor explaining variance in J. The addition of Fmax explained an additional 8.5% of the variance in proximal tibial J and 6.7% of the variance in midshaft tibial J, relative to Model 1. The addition of calf MCSA explained an additional 8.1% of the variance in proximal tibial J and 12% of the variance in midshaft tibial J, relative to Model 1.

## 4 DISCUSSION

This study aimed to determine what relationship estimated lower limb muscle force and power (proxied from thigh and calf MCSA and whole‐limb force plate mechanography) have with J quantified at commonly used section locations in bioarchaeology. Polar second moments of area at the midshaft femur, proximal tibia, and midshaft tibia were all strongly correlated with muscle size and function variables. However, these relationships between bone and muscle were patterned differently within the limb of athletes and controls, and reflected body size to differing extents. A summary of the main findings relevant to each research question is found in Table 7.

**TABLE 7:** Summary of main findings

Research question	Main findings	
	Athletes	Controls
Do estimates of muscle force and power influence femoral and tibial J?	Fmax significantly predicts J at all section locations.	
	Pmax never significantly predicts J.	
	Calf MCSA significantly predicts J at the proximal and midshaft tibia.	Thigh MCSA significantly predicts J at all three sections.
How does the relationship between J and MCSA pattern regionally within the lower limb?	Proximal and midshaft tibial J are more strongly correlated with thigh than calf MCSA	
	Midshaft femoral J is more strongly correlated with thigh than calf MCSA	
	Strongest significant correlation between MCSA and J is between thigh MCSA and the proximal tibia	Strongest significant correlation between MCSA and J is between thigh MCSA and the midshaft femur
Are estimated force (MCSAs and Fmax) and/or power variables, in combination with a proxy for lever arm length (stature), better independent predictors of variance in femoral and/or tibial J than stature and body mass alone?	Stature and either Fmax (all sections) or calf MCSA (tibial sections) explain significantly more variance in J than the base model (stature and body mass)	Stature and either Fmax or thigh MCSA (all sections) explain significantly more variance in J than the base model (stature and body mass).

### 4.1 Muscle force production is significantly associated with functionally related lower limb bone polar second moments of area

Our research sought to investigate the extent to which estimates of regional and/or whole‐limb muscle force and power influence femoral and tibial polar second moments of area, and the regional patterning of these relationships within the lower limb. Results found that, among both female athletes and controls, force estimates (both Fmax and MCSAs) consistently exhibited significant relationships with limb bone J even when controlling for body size. Whole‐limb Fmax exhibited stronger relationships with J in the tibia relative to the femur among both athletes and controls, suggesting that at least some of the variance in tibial J across the bioarchaeological literature may be reflecting the functional influence of muscle force‐generating capacity. The single‐leg hopping from which Fmax was quantified exposes the tibia in particular to maximal forces exerted by the main ankle plantarflexors, as well as the knee extensors to a lesser extent (Rantalainen et al., 2009; Sumnik et al., 2013; Veilleux & Rauch, 2010). The main ankle plantarflexors of the calf run parallel to the tibia, so during normal bipedal locomotion, they exert mainly compressive force on the diaphysis, particularly distally (Rittweger et al., 2000; Wehner, Claes, & Simon, 2009). However, as compressive load interacts with tibial diaphyseal curvature in more proximal section locations (Bertram & Biewener, 1988; Biewener, 1991; Capozza et al., 2010; Garcia & da Silva, 2004; Wehner et al., 2009), bending moments on the tibia increase. This may contribute to Fmax being a stronger predictor of J at the proximal rather than midshaft tibia.

The interpretation of lower limb bone J clearly requires the consideration of functional relationships with musculature. Among athletes, calf MCSA consistently maintained stronger relationships with J more distally, at the midshaft location (unadjusted *r* = .442; adjusted *r* = .423; *p* < .01), where it explained ~4% more of the variance in J than it did at the proximal tibia (12% vs. 8.1%), after accounting for stature. Among control subjects, thigh MCSA was a significant independent predictor of J more distally, at both the proximal and midshaft tibial J, explaining an additional 6% and 6.6% its variance, respectively, while calf MCSA was not. This pattern is similar to that documented by Rantalainen et al. (2013) among older adult males, whereby density‐weighted polar cross‐sectional moment of inertia (mg/cm) at the tibial midshaft were better predicted by thigh MCSA than calf MCSA (Rantalainen et al., 2013). Results of the current study may explain why earlier attempts to estimate muscle area from skeletal remains have had poor results: limb bone J is not necessarily strongly correlated with adjacent muscle areas from the same section location, but rather functionally related areas more proximally in the limb.

Despite being quantified in part from force measurements, Pmax consistently exhibited much weaker relationships with lower limb bone J than did Fmax, and never significantly explained any of the variance in J at any section location among either group of women once body size was accounted for. This follows expectations based on the findings of Hardcastle et al. (2014): Fmax influenced midshaft tibial geometry through the periosteal contour, and Pmax through the endosteal contour. Because J is most heavily influenced by periosteal change, J may not be a CSG property of bone that is informative about muscle power in life at our section locations. The sample composition of the athletes, including women with a range of athletic histories, may also be adding an additional source of variation that is known to affect peak power output: muscle conditioning (Hardcastle et al., 2014). For example, strength training has been shown to influence peak power output in the countermovement jump (Cormie et al., 2009). Certain types of training in particular, for example plyometrics (Holcolm, Lander, Rutland, & Wilson, 1996) or resistance training (McBride, Triplett‐McBride, Davie, & Newton, 2002), improve the capability to generate not just force but also acceleration, both crucial to maximizing power output. The variation in athletic history of the participants in this study introduces variation in experience and training that may be influencing their ability to generate power. In contrast, a variety of factors influence Fmax production, including kinematics, elastic properties of the Achilles tendon, and the biomechanical properties of the ankle and foot joints, all of which are less influenced by training and are relatively consistent across the lifespan (Hardcastle et al., 2014).

Interestingly, results of the current study also found that regional FCSAs were not related to lower limb bone J among control subjects, but they were among athletes, where both thigh and calf MCSAs were significantly negatively correlated with J throughout the lower limb. This likely reflects variation in the magnitude and duration of sport participation and the predominant ground impact loading characteristics among the athlete sample, rather than any direct functional relationship between fat and bone. Athletes who had participated intensively for many years in sport that exerts high ground impact loading on the lower limb have high polar second moments of area (Macintosh & Stock, 2019), and likely also have low fat mass relative to athletes with less intensive sporting histories.

### 4.2 Considering the interaction between muscle force and stature when interpreting cross‐sectional geometry

Our research also sought to investigate whether or not estimated force (MCSAs and Fmax) and/or power variables, in combination with a proxy for lever arm length (in this case, stature) are better independent predictors of variance in femoral and/or tibial J than stature and body mass alone. Stature and body mass together explained a substantial portion of the variance in J among control subjects, decreasing progressively from 71% at the midshaft femur to 53% at the midshaft tibia. This pattern supports bioarchaeological work documenting stronger correspondence between body size/breadth and lower limb bone CSG properties in the femur than tibia. However, this pattern was not documented among female athletes, where stature and body mass together exerted their strongest influence on J at the proximal tibia, explaining 37% of its variance. The major knee extensors and ankle plantarflexors make up a large component of body mass, and that musculature is functionally related to proximal tibial CSG. Thus, it may be that body mass itself is not particularly influential for shaping J among athletic women, but rather the muscular component of that mass (Pomeroy et al., 2018).

Though the nature of these relationships among males has yet to be determined, the results have implications for the way in which we deal with body size when attempting to interpret a behavioral signal from variation in lower limb bone J in the past. When muscle force was introduced into regression equations (either Fmax or MCSAs) for controls and athletes, it consistently replaced body mass as a significant factor explaining J, and body mass was not a significant predictive factor of midshaft femoral J at all among athletes. Together, it was an estimate of lever arm length (in this case, stature) and an estimate of muscle force (either Fmax or MCSAs) that explained the most variance in lower limb bone J, from 54 to 82% among controls and from 36 to 48% among athletes.

Until accurate methods of estimating muscle force from skeletal remains are developed, body mass still explains a significant component of variation in most lower limb section locations analyzed. However, results of the current study suggest that our potential to explain variation in midshaft diaphyseal bone strength parameters would be improved if we could (a) better estimate functionally relevant muscle force from bone, and (b) incorporate these force estimates alongside bone length into statistical analyses, rather than controlling for the effect of lever arm length as a component of body size standardization.

### 4.3 Implications for behavioral reconstruction

There are many components shaping the behavioral signal that we attempt to infer from variation in lower limb bone structural properties. The two groups of women in our study, athletes and control subjects, did not differ in bone‐related nutritional or hormonal status variables derived from questionnaire data, including age at menarche, family history of osteoporosis, dairy avoidance, past or current hormonal contraceptive use, age at first hormonal contraceptive use, and history of menstrual irregularity. Further, both athletes and controls were relatively homogeneous in age and ethnicity, none had a self‐reported history of eating disorder, all were premenopausal, and none had had children. The major differences between these two groups of women were related to loading characteristics, predominantly ground impact loading and muscle activity. In the absence of any significant lifetime ground impact loading, as was the case with the control group, from 63.7 to 81.6% of the variance in femoral and tibial J was accounted for just by a proxy for lever arm length (stature) and muscle force (Fmax), while body mass was not significant. This result suggests that variation in diaphyseal bone strength parameters may be better explained by the incorporation of a proxy for lever arm length (bone length in the case of skeletal remains) and improved estimates of muscle force from bone. Even among women with significant histories of impact loading associated with changes in their midshaft femoral and tibial J (Macintosh & Stock, 2019), 39.8 to 41.2% of the variance in femoral and tibial J was still accounted for just by a proxy for lever arm length (stature) and muscle force (Fmax). Results also demonstrate that the estimation of muscle force from limb bone cross‐sections should consider functional relationships between muscle and bone. For example, though midshaft femoral J exhibited stronger correlations with thigh MCSA obtained from the same section location than with less functionally related calf musculature distally in the limb, both proximal and midshaft tibial J exhibited stronger correlations with MCSAs from more proximal section locations. Further, though the midshaft tibial section location is utilized more commonly than the proximal 66% section location in behavioral inferences, the latter exhibits particularly strong relationships between muscle and bone among active individuals.

## 5 CONCLUSION

Lower limb bone polar second moments of area exhibited strong relationships with muscle force production, both estimated from mechanography and proxied by muscle areas, but not with power output. Muscle force, in combination with a proxy for lever arm length (in this case, stature), explained as much as 82% of the variance in lower limb bone J, and muscle force consistently replaced body mass as an independent predictor of variance in every instance. Results highlight the potential of better methods of estimating relevant muscle function variables (e.g., force and lever arm lengths) and their incorporation into statistical analyses for understanding behavioral signals inferred from skeletal remains. Potential functional relationships between musculature and limb bone diaphyseal J were documented, with particularly strong relationships between muscle force estimates and tibial J among athletes, and femoral J among controls. Future work on the interaction of musculature, size/proportions, and impact loading as interacting mechanisms shaping bone functional adaptation across a variety of contexts and locations in both the upper and lower limbs of both sexes is needed.

## AUTHOR CONTRIBUTIONS

**Alison Murray:** Conceptualization; data curation; formal analysis; investigation; methodology; writing‐original draft; writing‐review and editing. **Jay Stock:** Conceptualization; funding acquisition; methodology; resources; writing‐original draft; writing‐review and editing.

## Data Availability

The data that support the findings of this study are available on request from the corresponding author. The data are not publicly available due to privacy or ethical restrictions.
